# Establishment of visual assessment for the severity of dark circles in Chinese Han women

**DOI:** 10.1111/srt.13052

**Published:** 2021-05-17

**Authors:** Yimei Tan, Yanwen Jiang, Jiaping Chen, Hua Sun, Yuchen Qiu

**Affiliations:** ^1^ Skin & Cosmetic Research Department Shanghai Skin Disease Hospital Shanghai China; ^2^ Shanghai China‐norm Information Technology Co. Ltd Shanghai China

**Keywords:** dark circle, pigmented type, vascular type, visual assessment scale

## Abstract

**Objective:**

To establish a visual assessment scale for the severity of dark circles' pigmented and vascular type in Chinese women.

**Materials and method:**

A total of 269 healthy Chinese women from Shanghai with different degrees of dark circles', both pigmented and vascular types, were evaluated by visual assessment. Photographs of their dark circles were analyzed by image analysis.

**Results:**

The visual assessment evaluation on classification and severity showed a favorable agreement between the successive measured results. Significant differences from very slight to severe dark circles for pigmented type and vascular type were observed. The severity level by visual assessment was significantly positively correlated with ΔE values while negatively correlated with ΔL values (*P* < .01) in both pigmented and vascular types. Besides, Δa values of vascular type were significantly positively correlated with the ΔE values for dark circles' vascular type. Values between ΔE and ΔL also showed a significant negative correlation (*P* < .01).

**Conclusion:**

The five‐point visual assessment scale for dark circles of vascular and pigmented types was verified and proved to have good repeatability. The image analysis's objective result proved significant and consistent with the visual assessment and color parameters. This scale could be a useful and effective tool in diagnosing dark circles' severity.

## 1 INTRODUCTION

Dark circle is a common skin problem that affects individuals of any age where the surrounding skin around the eyes appears dark and dull.^1^ Dark circle is not a standard medical term but used by the public to indicate periorbital pigmentation. The contributions to dark circles are various. Multiple etiologic factors include dermal melanin deposition, postinflammatory hyperpigmentation secondary to atopic or allergic contact dermatitis, periorbital edema, superficial location of vasculature, and shadowing due to skin laxity.^2^, ^3^ According to its causes and definition, dark circles can be classified into vascular type, pigmented type, structural type, and mixed type.

The vascular type of dark circles is the predominant type in Asia, especially for Chinese people,^4^ notably caused by lack of sleep or fatigue, endocrine disorders, and anemia.^5^ Its physiology also involves inadequate blood supply around the eyes and blood stasis or retention causing skin cognition. Thinning of the lower eyelid can also make skin transparent, leading to the aggravated dark circle.^6^ Another common type is the pigmented type. The leading cause for its formation is excessive pigmentation, which is seen in dermal melanocytosis, allergic, and contact dermatitis caused by postinflammatory pigmentation.^6^, ^7^

Dark circle is a significant cosmetic concern. Patients, mostly women, are found to be commonly affected. Studies on dark circles show that a significant number of these patients were young adults between 20 and 30 years of age.^4^ They also complained of dissatisfaction with their facial appearance, fatigue, and insomnia. Lack of sleep was reported to be the major factor to dark circle in Asian population.^8^ Besides, stress or anxious, emotional liability, and aging can play a significant role in developing dark circles.^8^, ^9^ Although skin conditions under the eyes would not threaten physical health, dark circles influence facial appearance and make people look tired, sad, or lethargic, causing negative emotions. Hence, people invariably tend to seek treatment for this skin problem. More eye skincare products in the market to improve this problem.

Notably, the dark circle has gained more attention from consumers, especially women. Due to the individual basis and numerous causes, it is hard to define and classify dark circles. Previous studies have set up objective methods to measure dark circles according to their characteristics and causes.^5^, ^10^ Ultrasonography, wood's lamp, and image analysis were primarily applied for classification. Some studies have reported evaluating the degree of dark circles in general types.^10^ However, the assessment for the severity of an individual type of dark circle is yet to be reported. What's more, there is still lack of literature that focus on Chinese Han women although the problem of dark circle may be of greater concern in this population. It is reported the distribution of dark circle types varied among countries. Among Chinese population, vascular type with lighter skin (type I‐IV) is more frequent compared with other Asian population.^4^, ^8^ Pigmented type is another common type owing to excessive pigmentation under eyes in Asian population. It could be seen in conditions including dermal melanocytosis and postinflammatory hyperpigmentation.^6^ It presents irregular patches of brownish or gray pigmentation on the eyelids as a result of color transmission of gray or blue‐gray melanocytoses through the dermis.^4^, ^6^ Structural type of dark circle especially with tear trough is prevalent throughout 3 races in Asia, including Chinese, Malays, and Indians but less occurred among Chinese population.^8^ It is caused by shadowing which could be either tear trough or caused by skin laxity, puffy, or the overlying pseudoherniation of the infraorbital fat.^1^, ^11^ In this case, different angles of light could affect the appearance of dark circle. Thus, this study aimed to firstly establish a visual assessment scale to individually evaluate the severity of both pigmented and vascular types in Chinese women.

The established visual assessment scale will provide a supplement method for dark circles, which can also be applied in clinical research. The first step is to classify different types of dark circles. Notably, the primary symptom of dark circles is the change of color characteristic under the eyes. The dark circles were classified according to their color appearance by visual assessment to exclude an individual basis. The structural type of dark circles is excluded due to its structural shadows caused by skin contours. After classification, the severity level was scored based on the color difference between native skin tone and the periorbital skin. Photographs of dark circles were further analyzed by image analysis to verify the reliability between the color parameters obtained by image analysis and severity level by visual assessment.

## 2 MATERIALS AND METHOD

### 2.1 Subjects population

Two hundred sixty‐nine healthy Chinese women from Shanghai with different degrees of pigmented type or vascular type of dark circles were enrolled. The age ranged from 18‐60 years old. The dark circles were classified according to appearance characteristics through visual assessment. This study was approved by Shanghai institutional research ethics committee (Application Number: SECCR/2019‐38‐01) and all patients signed consent forms.

Before the evaluation, subjects were asked to wash their face using a standard cleanser and stay in a constant temperature‐controlled room for at least 30 minutes (20‐24°C, 40%‐60% room humidity). The photograph of the subjects’ front face was collected by VISIA‐CR (Canfield, Fairfield) under a standard light source (Mode 2). These photographs would be evaluated in the subsequent assessment.

### 2.2 Visual assessment

Experienced dermatologists evaluated the visual assessment of dark circles. Firstly, dark circles were classified into a pigmented or vascular type according to their clinical color appearance and causes.^5^, ^7^, ^12^ The vascular type of dark circle appears partially cyan, mainly located on the lower eyelid. The color will be lightened after pulling down the lower eyelid. This type has relatively thin eyelid skin with poor blood circulation, leading to hypoxia and microvascular blood veins' detention. It appears tawny and primarily located on the lower eyelid but, with the larger involved area for pigmented type. The color remains unchanged after pulling down the lower eyelid, which is associated with pigmented lesions. Reasons for UV stimulation that could accelerate the melanin synthesis or makeup residue are the triggering factors of this dark circle.

**FIGURE 1:**
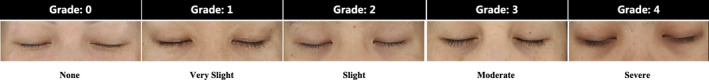
Grade of vascular type of severity grade

**FIGURE 2:**
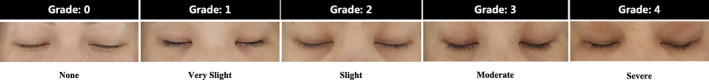
Grade of pigmented type of severity grade

According to previous studies, a new visual assessment scale was developed to evaluate the severity of pigment type and vascular type of dark circle regarding its color appearance based on dark circles classification. The proposed scale is a five‐point scale describing the color characteristic of vascular type (Figure 1) and pigmented type (Figure 2). The criteria are listed below:

Grade = 0, means none, without visible symptoms.

Grade = 1 means very slight. The color is light, and the involved area is limited, primarily located in the medial lower inner corner of the eyes.

Grade = 2 means slight. The color is light and covers the lower eyelid.

Grade = 3 means moderate. The color gets darker and cover both areas of the lower and upper eyelid,

Grade = 4 means severe. The color is darker and covers both lower and upper eyelids with the larger involved area. Besides, a half‐point score was applied to describe symptoms more accurately if its grade felled in between.

### 2.3 Image analysis

Images of dark circles from Visia‐CR were evaluated by the image analysis program (Image‐Pro Plus). The colors of dark circles (under eyes) and native skin tone (cheek) were measured. The results were reported as the L, a, and b color space. The color difference (ΔE) between the dark circles and the native skin tone was calculated using the equation below:

ΔE = $\sqrt{{\left(\Delta L\right)}^{2}+{\left(\Delta a\right)}^{2}+{\left(\Delta b\right)}^{2}}$

where ΔL, Δa, and Δb were the differences in the values of lightness (L), redness‐to‐greenness (a), and yellowness‐to‐blueness (b) between the dark circles and native skin tone.

### 2.4 Statistical analysis

The differences among groups of different degrees in each group were analyzed using one‐way ANOVA. Kappa test and intraclass correlation coefficient were used to evaluate test‐retest reliability. Spearman's *P*‐value was used to calculate the correlation between image analysis and visual assessment. Statistical analysis was performed using SPSS software (Version 24, IBM). A significant difference was measured at *P* < .05.

## 3 RESULT

### 3.1 Conformity assessment

Repeated evaluation of 100 images of the dark circle was conducted on the same computer screen to verify the reliability of visual assessment for dark circle images. The result showed that visual assessment had good repeatability of the classification and severity score. For the reliability of classification, the correlation coefficient (*r*) is .762 (*P* < .01), and for the severity score, *r* = .823 (*P* < .01).

### 3.2 Severity of dark circle evaluation by visual assessment and image analysis

The severity of dark circles was evaluated by a newly developed visual assessment scale and image analysis. Firstly, 269 subjects with different dark circles were classified into two types by the dermatologist according to the dark circles' definition and color appearance. The proportion of pigmented type is 45%, and vascular type is 55%.

**FIGURE 3:**
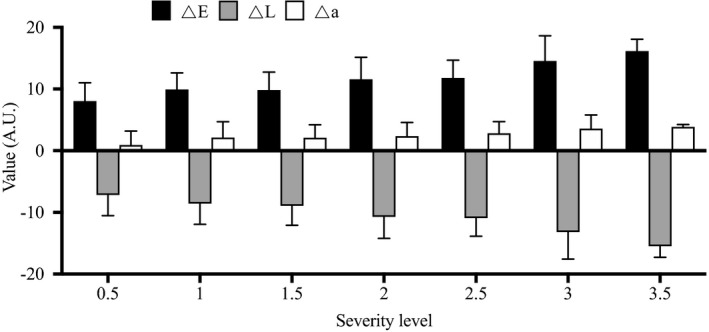
Changes in visual assessment and image analysis of vascular type of dark circles. ΔE, the color difference between dark circles and native skin tone; ΔL, the difference of lightness between dark circles and native skin tone; Δa, difference of redness‐to‐greenness between dark circles and native skin tone

By using the newly developed scale for dark circle severity with the range of 0‐4 score, it is found that there were significant differences from very slight to severe level of dark circles. In the result of vascular type, the most proportion was 52.4% from trim (slight) level (score 1.5 and 2), while others were 25.9% (very slight level; score 0.5 and 1), 20.8% (moderate level; score 2.5 and 3), and 1.0% (severe level; score 3.5). At the level of a severe degree, the highest score was 3.5. Notably, none of these subjects scored 4 (Table 1). The result of image analysis showed that the color difference values (ΔE) increased from 8.06 to 16.14, while the difference values of lightness (ΔL) decreased from −7.18 to 15.49 as the severity scores increased from 0.5 to 3.5 (Figure 3).

**TABLE 1:** Color parameters on the vascular type of dark circles among different severity levels (Mean ± SD)

Severity grade	ΔE	ΔL	Δa	Δb
0.5	8.06 ± 2.96	−7.18 ± 3.35	0.91 ± 2.26	−1.07 ± 2.13
1	9.94 ± 2.69	−8.58 ± 3.35	2.13 ± 2.57	−0.54 ± 3.17
1.5	9.82 ± 2.92	−8.94 ± 3.15	2.10 ± 2.12	−1.16 ± 2.26
2	11.59 ± 3.55	−10.72 ± 3.49	2.37 ± 2.21	−0.49 ± 3.04
2.5	11.81 ± 2.87	−10.92 ± 2.95	2.83 ± 1.90	−1.63 ± 2.38
3	14.54 ± 4.08	−13.18 ± 4.40	3.57 ± 2.22	−0.08 ± 4.28
3.5	16.14 ± 1.95	−15.49 ± 1.81	3.87 ± 0.39	1.30 ± 2.55

Note

ΔE, the color difference between dark circles and native skin tone; ΔL, the difference of lightness between dark circles and native skin tone; Δa, difference of redness‐to‐greenness between dark circles and native skin tone; Δb, the difference of yellowness‐to‐blueness between dark circles and native skin tone.

**FIGURE 4:**
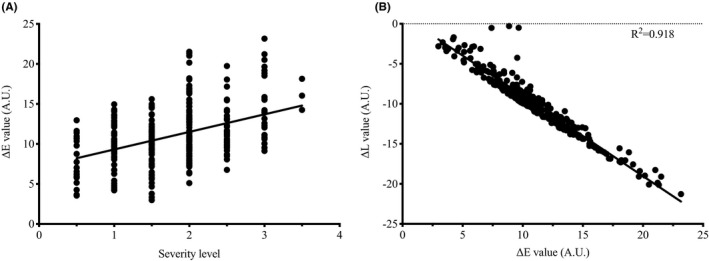
Correlations between severity level and color parameter values for vascular type. (A) Correlation between ΔE and severity level of vascular type of dark circles. (B) Correlation between ΔE values and ΔL values of vascular type of dark circles. ΔE, the color difference between dark circles and native skin tone; ΔL, the difference of lightness between dark circles and native skin tone

Furthermore, as shown in Figure 4A, the severity level was significantly positively correlated with ΔE values while negatively correlated with ΔL values (*P* <.01). Different redness‐to‐greenness values (Δa) also increased from 0.91 to 3.87 as the severity degree increased. Also, the Δa values of the vascular type of dark circle were significantly positively correlated with the ΔE values (*r* = .625, *P* < .01). Values between ΔE and ΔL also showed a significant negative correlation (*r* = .9179, *P* < .01) (Figure 4B).

**FIGURE 5:**
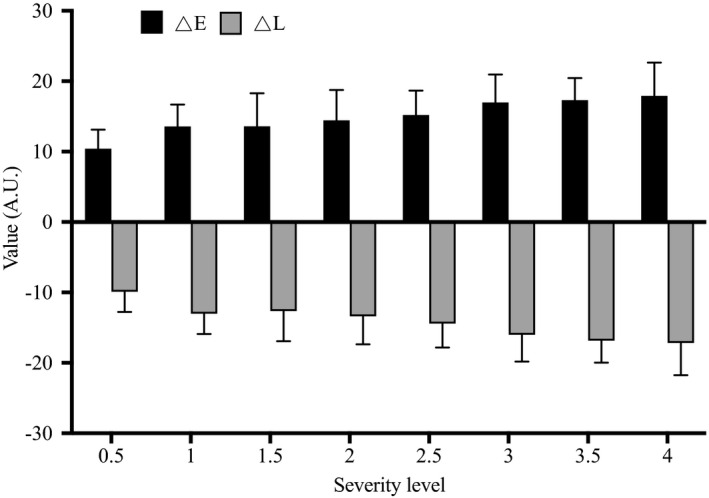
Changes in visual assessment and image analysis of the pigmented type of dark circles. ΔE, the color difference between dark circles and native skin tone; ΔL, the difference of lightness between dark circles and native skin tone

**FIGURE 6:**
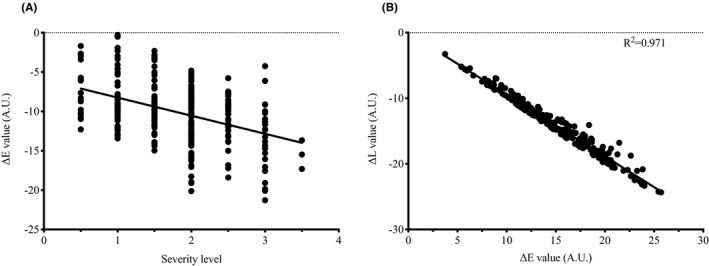
Correlation between severity level and color parameter values for pigmented type. (A) Correlation between ΔE and severity level of the pigmented type of dark circles. (B) Correlation between ΔE values and ΔL values of the pigmented type of dark circles. ΔE, the color difference between dark circles and native skin tone; ΔL, the difference of lightness between dark circles and native skin tone

For the severity level of pigmented type, significant differences in the ΔE and ΔL values between levels were observed (*P* < .05). The most are from the trim (slight) level (40.2%), which scored 1.5 and 2. The next is from a moderate level (35.7%), which scored 2.5 and 3. Others are 12.7% from a very slight level (score 0.5 and 1) and 11.5% from severe level (score 3.5 and 4) (Table 2). Image analysis showed that as the severity scores increased from 0.5 to 4, ΔE values significantly increased from 10.42 to 17.92, while ΔL values decreased from −9.91 to −17.18 (Figure 5). Interestingly, Figure 6A showed that the severity level of pigmented type by visual evaluation is significantly correlated with the ΔE and ΔL values (*P* < .01). Values between ΔE and ΔL also showed a significant negative correlation (*r* = .9174, *P* < .01) (Figure 6B).

**TABLE 2:** Color parameters on the pigmented type of dark circles among different severity levels (Mean ± SD)

Severity grade	ΔE	ΔL	Δa	Δb
0.5	10.42 ± 2.70	−9.91 ± 2.86	0.70 ± 1.16	−1.49 ± 2.47
1	13.57 ± 3.11	−13.02 ± 2.89	2.79 ± 1.77	0.29 ± 2.28
1.5	13.59 ± 4.70	−12.63 ± 4.29	2.73 ± 2.35	1.78 ± 3.59
2	14.45 ± 4.30	−13.39 ± 3.97	3.64 ± 2.20	1.34 ± 3.54
2.5	15.19 ± 3.48	−14.42 ± 3.39	3.00 ± 2.13	1.36 ± 2.87
3	16.98 ± 3.97	−16.03 ± 3.80	4.26 ± 1.99	1.09 ± 3.14
3.5	17.32 ± 3.13	−16.85 ± 3.12	3.18 ± 1.44	−0.07 ± 2.06
4	17.92 ± 4.74	−17.18 ± 4.58	1.96 ± 1.52	−2.54 ± 3.97

Note

ΔE, the color difference between dark circles and native skin tone; ΔL, the difference of lightness between dark circles and native skin tone; Δa, difference of redness‐to‐greenness between dark circles and native skin tone; Δb, the difference of yellowness‐to‐blueness between dark circles and native skin tone.

## 4 DISCUSSION

Despite the significant prevalence of dark circles, it remained an unsolved and complex problem to diagnose this clinical manifestation. Different types of dark circles have their unique characteristics.^4^ We proposed that it is necessary to evaluate each type with different assessment scales. As far as we know, reported articles focused more on the classification of dark circles, and the assessment scale for severity level was used for all types. Thus, this newly developed visual assessment scale was proposed to evaluate the severity level for pigmented type and vascular type of dark circles more effortlessly and intuitively. The visual evaluation scale has validated against image analysis by IPP software and could effectively discriminate different dark circles' severity levels. Among the 269 enrolled Chinese women in this study, it was found that dark circles in Chinese women are characterized by significant darker color on eyelids. Image analysis results showed that the severity levels of visual assessment for both pigmented type and vascular type of dark circles had significant positive correlations with ΔE values (*P* < .01) and significant negative correlations with ΔL values (*P* < .01). It suggested that the severe the circles, the much darker and the less light‐toned, the lower eyelid would be. Also, the lightness of skin around eyes significantly contributed to dark circles' appearance according to the significant negative correlations between ΔE and ΔL values for both vascular and pigmented types of dark circles (Figures 2 and 3). In evaluating the vascular type of dark circles, it is found that Δa values are significantly positively related to the ΔE values. Clinically, stasis or congestion of cutaneous blood in the lower eyelid is the primary cause of dark circles' vascular type. Dermis in the eyelid displays a lower amount of collagen, elastin, and glycosminoglycan than dermis at other sites,^13^ presented a thinner eyelid suggested by ultrasound images analysis.^14^ In this case, it was proposed that the skin color of lower eyelid appeared much redder in the vascular type of dark circle due to the clogged cutaneous blood and thinner eyelids as the severity level increased.

However, the proposed scale still has some weaknesses. Firstly, some fluctuations were observed in the color parameters (ΔE and ΔL), especially at the half‐point level. Table 1 and Table 2 show that the visual assessment and color parameters presented a significant correlation in the overall trend. ΔE and ΔL values at the half‐point level are not significantly different from those at adjacent integral levels. In this case, one should be careful when using the half‐point to evaluate the severity level. Larger sample size is needed to refine the half‐point level and precisely characterize it.

In general, this visual assessment scale provided a straightforward method to evaluate the vascular and pigmented types of dark circles. Compared with published methods, the present study can be done more intuitively and conveniently without using an expensive instrument and complex analysis. Reduced lightness and increased color differences are the primary characteristics of dark circles for pigmented and vascular types among Chinese women. Notably, the visual assessment was verified and supported by objective image analysis, demonstrating it is repeatable and reasonable. Significant correlation between visual assessment and image analysis was also observed. This scale, combined with image analysis, could be a useful and effective tool in diagnosing the severity of dark circles for both vascular and pigmented types.

## 5 CONCLUSIONS

A five‐point visual assessment scale for dark circles was developed based on vascular and pigmented types' color characteristics. It is proposed that this new visual assessment scale can be applied to the clinical research and efficacy evaluation for cosmetic products to remove dark circles, though half‐point scale is still needed to be refined in our further study. Besides, the mixed type, which is a combination of vascular and pigmented type, is also common among woman consumers.^10^ This type is frequent in Chinese population only secondary to vascular type.^8^ Considering its multifactorial etiology and high prevalence, we are making effort to explore further the characteristics of mixed type of dark circle with larger sample size in order to optimize this visual assessment scale. Next, the scale of the evaluation for the severity of mixed type of dark circle would be established based on the characteristics and refined visual scale of pigmentary and vascular type. Thus, more studies are needed to broaden our knowledge of this (cosmetic) condition.

## CONFLICT OF INTEREST

This study is conducted based on clinical tests and has got permission from sponsors. The results were only used for research. All authors declared there was no conflict of interest.
